# Thalamocortical connectivity and nucleus-specific thalamic dysfunction in temporal lobe epilepsy: an integrative review

**DOI:** 10.1007/s00429-026-03169-7

**Published:** 2026-08-04

**Authors:** Giuseppina Bevacqua, Marco Ciavarro, Lorenzo Gaetani, Valentina Grespi, Carlo Conti

**Affiliations:** 1 University of Perugia, Perugia, Italy; 2 Department of Neuroscience, Neurosurgery Unit, Santa Maria Hospital, Terni, Italy; 3 Department of Neurosurgery, IRCCS Neuromed, Pozzilli, IS Italy; 4 Department of Neuroscience, Neurology Unit, Perugia University Hospital, Perugia, Italy; 5 Stem Cell Laboratory, Cell Factory and Biobank Santa Maria Hospital, Terni, Italy; 6 Neurosurgery Unit, Perugia University Hospital, Perugia, Italy

**Keywords:** Temporal lobe epilepsy, Thalamocortical networks, fMRI, Graph theory, Network topology

## Abstract

Temporal lobe epilepsy (TLE) is increasingly recognized as a distributed network disorder extending beyond mesial temporal structures. Among the brain regions implicated in epileptic network organization, the thalamus has emerged as a key hub due to its extensive structural and functional connections with cortical, limbic, and subcortical systems.This integrative review aimed to synthesize current evidence regarding nucleus-specific thalamic abnormalities and thalamocortical connectivity alterations in TLE.A literature search was conducted following PRISMA guidelines using PubMed and Google Scholar databases. Twenty-eight studies were included in the qualitative synthesis, with nineteen studies providing detailed nucleus-specific structural, functional, or electrophysiological characterization of thalamic involvement.Across structural MRI, diffusion imaging, resting-state functional MRI, magnetoencephalography, and stereo-electroencephalography studies, convergent evidence indicated preferential involvement of the anterior thalamic nucleus (ANT), mediodorsal nucleus (MD), pulvinar, and intralaminar nuclei. TLE with mesial temporal sclerosis generally exhibited more extensive thalamic atrophy and thalamotemporal disconnection, whereas MRI-negative TLE showed more heterogeneous functional and network-level abnormalities. Electrophysiological studies demonstrated dynamic thalamic participation in seizure propagation and network synchronization, supporting an active role of thalamic nuclei in epileptic processes. Emerging longitudinal evidence further suggests that thalamocortical networks may undergo postoperative reorganization following successful epilepsy surgery.Overall, the available evidence supports a transition from a predominantly hippocampocentric framework toward a distributed thalamocortical network model of TLE. Nucleus-specific thalamic dysfunction appears to contribute to seizure propagation, cognitive impairment, and large-scale network dysregulation, highlighting the importance of multimodal approaches for understanding epileptic network organization.

## Introduction

Temporal lobe epilepsy (TLE) represents the most common focal epilepsy syndrome in adults and is increasingly recognized as a disorder involving distributed brain networks rather than an isolated hippocampal pathology (King et al. 1996; Blümcke et al. 2013; González Otárula and Schuele 2020; Whelan et al. 2018). While mesial temporal structures—including the hippocampus, entorhinal cortex, and parahippocampal regions—remain central components of the epileptogenic system, growing evidence suggests that seizure generation, propagation, cognitive dysfunction, and treatment response emerge from alterations affecting broader cortico-subcortical circuits (Tatum 2012; González Otárula and Schuele 2020; Bernhardt et al. 2012).

Among these networks, the thalamus has gained increasing attention because of its extensive reciprocal connectivity with cortical, limbic, and basal ganglia structures (Guye et al. 2006; He et al. 2020). Rather than acting as a passive relay station, the thalamus contributes to large-scale information integration, regulation of cortical synchrony, attentional control, and memory processing (Sherman and Guillery 2002; He et al. 2020). These functions position thalamic nuclei as plausible modulators of epileptic activity and potential contributors to seizure propagation and cognitive impairment in TLE (Guye et al. 2006; Li et al. 2021; Pang et al. 2024).

Recent advances in structural neuroimaging, diffusion-based tractography, resting-state functional MRI, graph-theoretical analyses, magnetoencephalography (MEG), and stereo-electroencephalography (SEEG) have substantially improved the characterization of thalamocortical networks in epilepsy (Keller et al. 2014; Li et al. 2021; Chen et al. 2024; Zhang et al. 2025; Soulier et al. 2023). In parallel, increasing clinical interest in neuromodulatory approaches targeting thalamic nuclei—including anterior thalamic stimulation—has further emphasized the importance of understanding nucleus-specific thalamic contributions to epileptic networks (Li et al. 2021; Soulier et al. 2023).

Accumulating evidence suggests that distinct thalamic nuclei demonstrate differential vulnerability across TLE phenotypes (Mueller et al. 2009; Yildirim et al. 2025). The anterior thalamic nucleus (ANT), mediodorsal nucleus (MD), pulvinar, and intralaminar nuclei have each been implicated in specific aspects of seizure dynamics, functional network organization, and cognitive dysfunction (Tung et al. 2023; Li et al. 2021; Pang et al. 2024). Moreover, emerging data indicate that temporal lobe epilepsy with mesial temporal sclerosis (TLE-MTS) and MRI-negative temporal lobe epilepsy (TLE-MRIneg) may exhibit distinct patterns of thalamic structural and functional involvement, suggesting subtype-specific network phenotypes (Mueller et al. 2009; Yildirim et al. 2025).

Although multiple studies have independently characterized structural abnormalities, white matter disconnection, altered functional connectivity, and electrophysiological changes involving the thalamus in TLE (Keller et al. 2014; Guye et al. 2006; Pang et al. 2024), an integrated synthesis linking these findings across organizational levels remains limited. Specifically, the extent to which structural alterations align with functional disturbances, whether nucleus-specific patterns emerge consistently across modalities, and how these findings inform network models of temporal lobe epilepsy remain incompletely understood.

In this review, structural connectivity refers to anatomical pathways connecting thalamic and cortical regions, primarily assessed through diffusion-based neuroimaging approaches, whereas functional connectivity refers to statistical dependencies between neural signals measured through modalities such as functional MRI, electrophysiology, and MEG. Thalamic dysfunction is considered here as encompassing structural abnormalities, altered connectivity patterns, and electrophysiological alterations contributing to pathological network organization.

The aim of this review is therefore to synthesize current evidence regarding structural, functional, and electrophysiological alterations involving thalamic nuclei in temporal lobe epilepsy, with particular emphasis on nucleus-specific contributions, subtype-dependent network phenotypes, and emerging systems-level models of thalamocortical dysfunction.

## Methods

### Search strategy

This integrative review was conducted following the Preferred Reporting Items for Systematic Reviews and Meta-Analyses (PRISMA) framework for literature identification, screening, and reporting. A systematic literature search was performed using PubMed and Google Scholar to identify studies investigating thalamic structural abnormalities, structural connectivity, functional connectivity, and electrophysiological alterations in temporal lobe epilepsy (TLE).

The search included studies published between January 1996 and November 2025. Database searches were independently conducted by the authors and complemented through manual screening of references from eligible articles to identify additional relevant studies.

Search terms combined Medical Subject Headings (MeSH) and free-text terms and included:

(“Temporal Lobe Epilepsy” OR “TLE”) AND (“Thalamus” OR “Thalamic nuclei”) AND (“Connectivity” OR “Structural Connectivity” OR “Functional Connectivity” OR “Diffusion Tensor Imaging” OR “Resting-state fMRI” OR “SEEG” OR “Stereo-electroencephalography” OR “Magnetoencephalography” OR “Network”).Only English-language studies were considered eligible. Given the methodological heterogeneity and the integrative nature of the review, a formal risk-of-bias assessment was not performed.

### Eligibility criteria

Studies were included when they met the following criteria:

- original peer-reviewed human studies involving patients with temporal lobe epilepsy;
- studies investigating structural, diffusion-based, functional, metabolic, or electrophysiological thalamic alterations;
- studies reporting thalamic nuclei involvement or thalamocortical network findings;
- studies providing sufficient methodological and clinical information for interpretation.

Studies were excluded when they:

- focused exclusively on extra-temporal epilepsy syndromes;
- consisted of reviews, editorials, letters, conference abstracts, or isolated case reports;
- exclusively involved animal models;
- lacked specific information regarding thalamic involvement;
- reported insufficient methodological characterization.

Studies involving mixed epilepsy populations were retained only when temporal lobe epilepsy-specific findings could be separately extracted.

### Study selection

After duplicate removal, titles and abstracts were screened for eligibility.

Potentially relevant studies subsequently underwent full-text assessment according to predefined inclusion and exclusion criteria.

Study selection was independently performed by the authors, and disagreements were resolved through discussion and consensus.

**Fig. 1:**
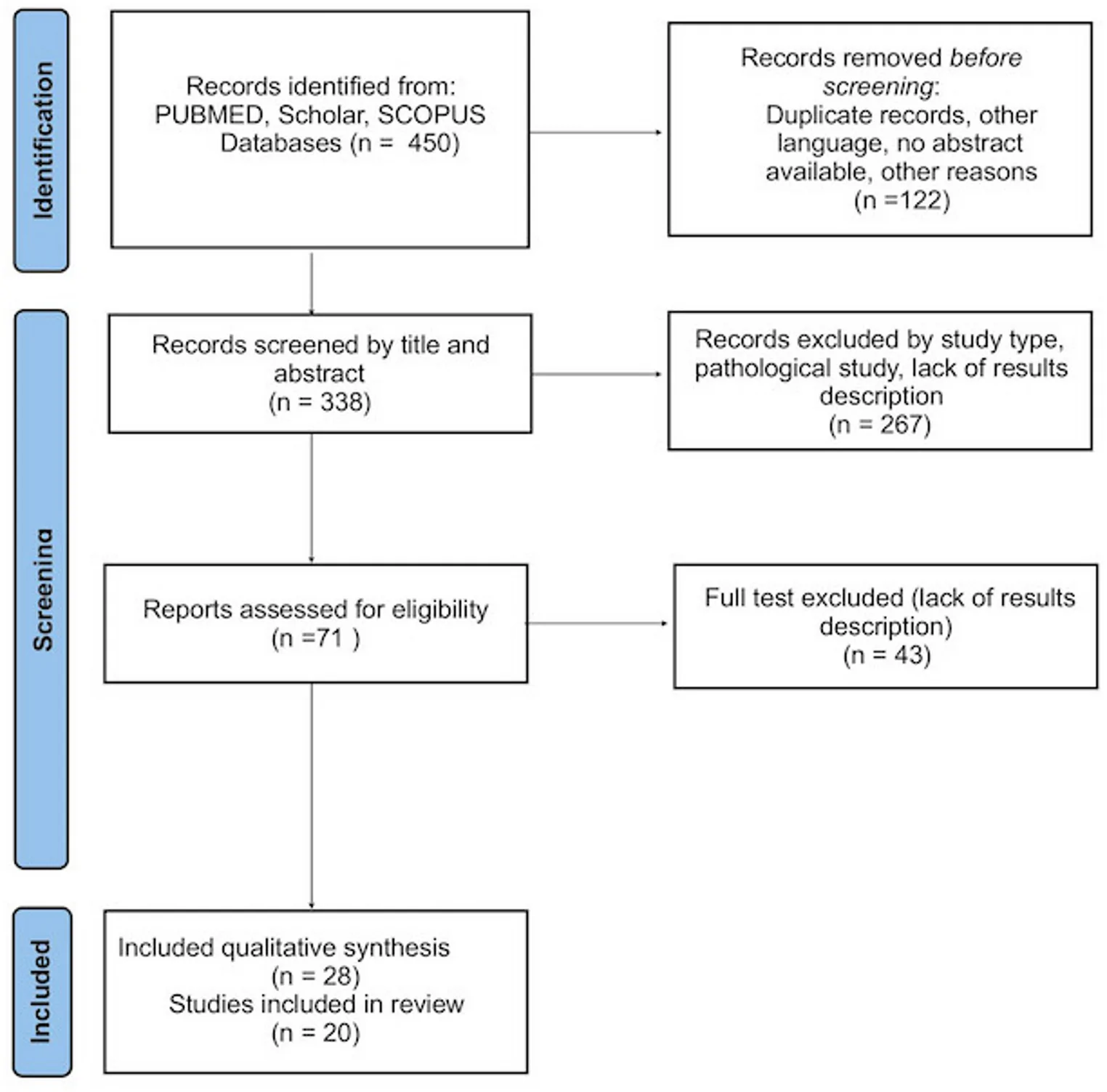
A PRISMA flow diagram showing the flow of information through the different phases of the systematic review

The study identification and selection process is summarized in the PRISMA flow diagram (Fig. 1) (Shamseer et al. 2015).

### Data extraction

The following variables were extracted from eligible studies:

- study design;
- sample size;
- temporal lobe epilepsy subtype;
- imaging or electrophysiological modality;
- thalamic nuclei investigated;
- principal structural, functional, or metabolic findings;
- connectivity and network-level observations.

Particular emphasis was placed on identifying convergent findings, discordant evidence, and methodological heterogeneity across studies.

### Evidence synthesis

Given the substantial heterogeneity of study populations, imaging modalities, electrophysiological approaches, analytical pipelines, and reported outcome measures, quantitative synthesis was not feasible.

A qualitative integrative synthesis approach was therefore adopted. Findings were organized according to:

1. structural thalamic alterations;
2. structural connectivity abnormalities;
3. functional connectivity alterations;
4. electrophysiological findings;
5. network-level organization and systems-level integration.

Particular attention was given to differences between temporal lobe epilepsy with mesial temporal sclerosis (TLE-MTS) and MRI-negative temporal lobe epilepsy (TLE-MRIneg), as well as to convergent versus discordant findings across modalities.

Following screening and eligibility assessment, 28 studies were included in the qualitative synthesis. Among these, 20 studies provided detailed nucleus-specific structural, functional, or electrophysiological characterization of thalamic involvement and therefore constituted the core studies included in the integrative analysis presented in the Results section. Their main characteristics are summarized in Table 1.

Additional studies reporting partial volumetric, diffusion, metabolic, or network-level findings without comprehensive nucleus-specific characterization were included to contextualize convergent and discordant evidence across modalities.

**Table 1:** Characteristics of included studies investigating thalamic abnormalities and thalamocortical connectivity in temporal lobe epilepsy

Study	*N*	TLE subtype	Modality	Thalamic nuclei	Main findings
Guye et al. (2006)	13	TLE	SEEG	ANT	Early thalamic recruitment during seizure propagation
Mueller et al. (2009)	37	TLE-MTS/MRI neg	MRI volumetry	ANT, LD	ANT atrophy in TLE-MTS
Bernhardt et al. (2012)	26	TLE-MTS	Structural MRI	MD, pulvinar	Thalamic atrophy associated with cortical thinning and disease duration
Keller et al. (2014)	23	TLE-MTS	DTI	ANT	Reduced thalamotemporal FA
Barron et al. (2014)	19	TLE-MTS	MRI volumetry /DTI	Pulvinar	Volumetric reduction in the thalamic-hippocampal connections
Výtvarová et al. (2016)	22	TLE	rs-fMRI, graph theory		Mesio-temporal connectivity is more severely impaired in left TLE
Chiosa et al. (2017)	12	TLE	EEG/MRI volumetry	ANT, MD, pulvinar	Reduced thalamic volume. Altered connectivity with anterior, lateral, and pulvinar nuclei
Whelan et al. (2018)	2149	MTLE-L/MTLE-R	Multicenter structural MRI	Bilateral thalamus	Bilateral subcortical volume reduction supporting network-level involvement
Assenza et al. (2020)	12	TLE	HF-SEP	ANT, CL, VPM	Prolonged HF-SEP duration in the epileptic hemisphere, suggesting impaired thalamocortical signal integration and interictal network dysfunction
He et al. (2020)	96	TLE	Functional connectivity analysis	Thalamocortical loops	Disrupted cortico-basal ganglia-thalamic organization
Wills et al. (2021)	40	TLE-MTS	Resting-state fMRI + ALFF	Lateral pulvinar, CL	Reduced PuL–lateral occipital connectivity and altered CL–medial occipital connectivity
Rong Li et al. (2021)	26	TLE	Resting-state fMRI/Seed-based functional connectivity	ANT, CL, VPM	Reduced connectivity between ANT and hippocampal pathways associated with memory impairment
Li et al. (2022)	24	TLE	rs-fMRI, static FC, dynamic FC, dynamic effective connectivity	Pulvinar, ANT	Altered dynamic synchronization and seizure-related thalamic recruitment
Tung et al. (2022, 2023)	75	Left vs. right TLE	MRI volumetry /PET	ANT, Medial and posterior nuclei, pulvinar	Nucleus-specific asymmetrical thalamic involvement
Soulier et al. (2023)	6	TLE-MTS	SEEG	ANT, Pulvinar	Progressive thalamic participation during seizure evolution
Pang et al. (2024)	53	TLE	rs-fMRI parcellation	ANT, MD	Reduced modular segregation and altered hub organization
Chen et al. (2024)	35	TLE	Graph-theoretical fMRI	Thalamic subnetworks	Abnormal hub centrality and increased large-scale synchronization
Li et al. (2025)	76	Postoperative TLE	Longitudinal MRI/rs-fMRI	Thalamocortical networks	Partial postoperative restoration of connectivity and network remodeling
Yildirim et al. (2025)	36	TLE-MTS/TLE-MRIneg	High-resolution MRI and tractography	ANT, Medial thalamic nuclei	Reduced hippocampus–ANT connectivity and subtype-specific thalamic abnormalities
Zhang et al. (2025)	34	TLE	MEG	Pulvinar	Abnormal alpha- and beta-band thalamocortical synchronization

TLE-MTS: temporal lobe epilepsy with mesial temporal sclerosis; TLE-MRIneg: MRI-negative temporal lobe epilepsy; ANT: anterior thalamic nucleus; MD: mediodorsal nucleus; CL: central lateral nucleus; LD: lateral dorsal nucleus; VPM: Ventral posteromedial nucleus; DTI: diffusion tensor imaging; rs-fMRI: resting-state functional MRI; SEEG: stereo-electroencephalography; MEG: magnetoencephalography; ALFF: FMRI amplitude of low frequency fluctuation; HF- SEP: high frequency oscillations of somatosensory evoked potentials ; FC: functional connectivity

## Results

### Structural thalamic alterations

Across structural MRI investigations, convergent evidence indicates preferential involvement of anterior, mediodorsal, and pulvinar thalamic nuclei in temporal lobe epilepsy, although the extent and distribution of abnormalities vary according to epilepsy subtype, disease duration, and lateralization.

#### TLE-MTS

Studies involving patients with temporal lobe epilepsy and mesial temporal sclerosis (TLE-MTS) consistently demonstrated widespread thalamic atrophy, particularly involving the anterior thalamic nucleus (ANT), mediodorsal nucleus (MD), and pulvinar (Mueller et al. 2009; Bernhardt et al. 2012; Keller et al. 2014; Yildirim et al. 2025). Structural alterations were frequently associated with hippocampal CA1 atrophy, limbic cortical thinning, and disease duration, supporting the concept of progressive network-level degeneration extending beyond mesial temporal structures.

More recent high-resolution parcellation studies reinforced the preferential vulnerability of the ANT in TLE-MTS. Yildirim et al. (2025) demonstrated marked ipsilateral and bilateral ANT atrophy accompanied by reduced hippocampus–ANT structural connectivity. These alterations correlated with epilepsy duration and seizure burden, suggesting progressive disruption of thalamotemporal pathways over time.

Large-scale structural analyses further support widespread network involvement. ENIGMA-Epilepsy investigations and additional volumetric MRI studies reported bilateral subcortical abnormalities extending beyond the epileptogenic temporal lobe, reinforcing the concept of TLE as a distributed network disorder rather than a focal hippocampal pathology (Whelan et al. 2018; Bernhardt et al. 2012).

#### TLE-MRIneg

Compared with TLE-MTS, MRI-negative temporal lobe epilepsy (TLE-MRIneg) generally demonstrated more restricted and heterogeneous thalamic abnormalities. Mueller et al. (2009) identified limited involvement of lateral dorsal nuclei, whereas Yildirim et al. (2025) observed preferential medial thalamic involvement with relative sparing of the anterior nucleus.

These findings suggest that TLE-MRIneg may represent a distinct network phenotype characterized by less extensive structural degeneration but altered thalamotemporal organization. However, evidence remains comparatively limited and heterogeneous across cohorts, likely reflecting variability in patient selection, imaging methodology, and underlying epileptogenic substrates.

#### Laterality-related findings

Several studies identified asymmetrical thalamic involvement according to seizure lateralization. Left-sided TLE was generally associated with more pronounced thalamic atrophy and metabolic dysfunction, particularly involving medial and posterior thalamic nuclei (Tung et al. 2022, 2023).

Functional and structural network studies additionally reported reduced subcortical integration and altered hub organization that appeared more extensive in left temporal lobe epilepsy compared with right-sided disease (Výtvarová et al. 2016). These findings suggest potential hemispheric differences in thalamocortical network organization and vulnerability.

### Structural connectivity abnormalities

Diffusion tensor imaging and tractography studies consistently demonstrated disruption of thalamotemporal white matter pathways in temporal lobe epilepsy, although the anatomical distribution and severity of abnormalities varied substantially across studies.

Reduced fractional anisotropy and increased mean diffusivity within thalamotemporal pathways were frequently observed in TLE-MTS and were associated with executive dysfunction, disease duration, and widespread limbic network abnormalities (Keller et al. 2014; Law et al. 2018; Kim et al. 2010). Several investigations additionally reported bilateral white matter alterations despite unilateral seizure onset, supporting the concept of diffuse network involvement (Concha et al. 2010, Bertram and Zhang 1999).

High-resolution tractography studies further highlighted subtype-specific patterns of disconnection. Yildirim et al. (2025) demonstrated reduced hippocampus–ANT structural connectivity in TLE-MTS, whereas TLE-MRIneg showed more limited alterations involving ventral and medial thalamic pathways.

Despite convergent evidence suggesting thalamotemporal disconnection, important methodological heterogeneity exists across diffusion studies, including variability in tract reconstruction methods, parcellation strategies, and patient selection criteria.

### Functional connectivity alterations

#### Resting-state functional connectivity

Resting-state functional MRI studies consistently demonstrated altered thalamocortical functional connectivity in temporal lobe epilepsy, particularly involving limbic, sensorimotor, default mode, and prefrontal networks (Li et al. 1996).

Reduced connectivity between the ANT and hippocampal regions represented one of the most reproducible findings across studies (Li et al. 2021; Pang et al. 2024). Additional investigations identified altered integration involving mediodorsal and pulvinar nuclei, suggesting widespread disruption of thalamic hub organization within large-scale cortical networks.

Graph-theoretical analyses further demonstrated reduced modular segregation, altered hub centrality, and increased large-scale synchronization involving thalamic subnetworks (He et al. 2020; Chen et al. 2024). However, the directionality of connectivity changes was not uniform across studies, with some investigations reporting increased thalamocortical integration potentially reflecting maladaptive synchronization or compensatory reorganization.

#### Dynamic functional connectivity

Dynamic connectivity analyses suggested that thalamic dysfunction in TLE is temporally variable and state-dependent rather than static.

Frequency-specific electrophysiological and imaging studies demonstrated reduced variability of thalamocortical interactions, altered alpha-band connectivity, and transient increases in temporal–thalamic synchronization preceding epileptic discharges (Chiosa et al. 2017; Li et al. 2022).

SEEG investigations additionally revealed dynamic changes across seizure evolution, characterized by early cortical-to-thalamic recruitment followed by progressive thalamic participation during seizure propagation and termination (Guye et al. 2006; Soulier et al. 2023).

#### Compensatory network reorganization

Not all functional alterations appeared maladaptive. Several studies identified increased connectivity involving prefrontal, posterior cingulate, and precuneus regions, potentially reflecting compensatory network recruitment aimed at preserving cognitive function (Výtvarová et al. 2016; Li et al. 2021, 2022).

These findings suggest coexistence of pathological synchronization and adaptive network plasticity within thalamocortical systems.

### Electrophysiological evidence

Electrophysiological investigations further support a central role of thalamic nuclei in seizure propagation, synchronization, and termination in temporal lobe epilepsy. Evidence derived from stereo-electroencephalography (SEEG), magnetoencephalography (MEG), and evoked potential studies indicates that thalamic involvement is dynamic and evolves throughout seizure progression rather than representing a static network abnormality.

SEEG studies demonstrated that the anterior thalamic nucleus (ANT) frequently becomes recruited during seizure propagation, particularly in mesial temporal lobe epilepsy, where thalamic activation may occur rapidly after hippocampal involvement (Guye et al. 2006; Soulier et al. 2023). These findings are consistent with the hypothesis that the ANT participates in large-scale limbic synchronization and contributes to propagation within temporolimbic circuits.

Additional electrophysiological evidence suggests differential functional roles across thalamic nuclei. While ANT recruitment appears closely associated with seizure propagation and synchronization, pulvinar activation has been linked to seizure termination and modulation of posterior cortical synchronization patterns (Li et al. 2022; Zhang et al. 2025). This nucleus-specific functional differentiation reinforces the concept that thalamic contributions to epilepsy are not homogeneous but instead depend on distinct anatomical and functional network affiliations.

MEG studies additionally identified abnormal frequency-dependent thalamocortical synchronization, particularly involving alpha- and beta-band oscillatory activity (Zhang et al. 2025). Altered synchronization patterns were associated with impaired network segregation and increased large-scale coupling, suggesting abnormal regulation of cortical excitability.

High-frequency somatosensory evoked potential studies further demonstrated altered thalamocortical transmission and impaired inhibitory modulation in TLE patients, supporting the presence of widespread subcortical dysfunction extending beyond the epileptogenic temporal lobe (Assenza et al. 2020).

Collectively, electrophysiological findings are consistent with the concept of the thalamus as an active participant in seizure dynamics rather than a passive relay structure. However, important heterogeneity remains regarding the timing, laterality, and nucleus-specific patterns of thalamic recruitment across studies.

### Postoperative thalamic remodeling

Emerging longitudinal evidence suggests that thalamic abnormalities in temporal lobe epilepsy may demonstrate partial postoperative reorganization following successful surgical treatment.

Recent imaging studies reported progressive changes in thalamocortical connectivity and network topology after temporal lobectomy, indicating that at least part of the observed thalamic dysfunction may reflect dynamic network reconfiguration rather than irreversible structural damage alone (Li et al. 2025). Postoperative reductions in pathological synchronization and partial restoration of functional connectivity were particularly observed within limbic and default mode networks.

These findings are consistent with the hypothesis that epileptic activity contributes to progressive network-level remodeling involving both cortical and subcortical structures. Surgical interruption of epileptogenic pathways may therefore promote adaptive reorganization extending beyond the temporal lobe itself.

Nevertheless, postoperative studies remain limited in number and frequently involve relatively short follow-up periods and heterogeneous patient populations. Moreover, the extent to which postoperative normalization depends on seizure freedom, disease duration, or preoperative network integrity remains incompletely understood.

Further longitudinal multimodal investigations are required to clarify whether thalamic abnormalities primarily represent irreversible neurodegenerative changes, compensatory adaptations, or dynamic seizure-related network alterations.

### Network-level integration and systems-level interpretation

Across structural, diffusion-based, functional, and electrophysiological modalities, the available evidence consistently supports the conceptualization of temporal lobe epilepsy as a distributed cortico-subcortical network disorder involving nucleus-specific thalamic dysfunction.

Convergent findings across studies suggest preferential involvement of the anterior thalamic nucleus, mediodorsal nucleus, and pulvinar within partially overlapping but functionally distinct epileptic subnetworks. Structural atrophy, white matter disconnection, abnormal synchronization, and altered hub organization were consistently observed across modalities. Collectively, these findings support disruption of large-scale thalamocortical integration.

Importantly, several studies suggest that thalamic abnormalities extend beyond direct hippocampal connectivity and involve broader cortico-basal ganglia-thalamic loops (He et al. 2020). These systems-level alterations may contribute not only to seizure propagation but also to cognitive dysfunction, impaired attentional regulation, and reduced network adaptability observed in TLE patients.

Graph-theoretical investigations further suggest the hypothesis that the thalamus functions as a critical connector hub within epileptic networks. Altered hub centrality, reduced modular segregation, and abnormal large-scale synchronization may facilitate propagation of epileptic activity across distributed cortical systems (Chen et al. 2024; Pang et al. 2024).

At the same time, substantial methodological and biological heterogeneity remains across studies. Variability in imaging pipelines, parcellation strategies, epilepsy subtype classification, seizure lateralization, disease duration, and medication exposure likely contributes to inconsistencies regarding the extent and laterality of thalamic abnormalities.

Differences between TLE-MTS and TLE-MRIneg appear particularly relevant. While TLE-MTS generally demonstrates more extensive structural degeneration and thalamotemporal disconnection, MRI-negative TLE may exhibit more subtle but functionally significant network alterations. These observations suggest that distinct TLE phenotypes may involve partially different mechanisms of thalamocortical dysfunction.

Overall, the available evidence suggests a transition from a predominantly hippocampocentric framework toward a distributed network model in which nucleus-specific thalamic dysfunction contributes to epileptogenic organization, seizure propagation, and large-scale network dysregulation in temporal lobe epilepsy.

## Discussion

### From hippocampocentric to distributed network models of temporal lobe epilepsy

Traditionally, temporal lobe epilepsy has been conceptualized primarily as a disorder centered on mesial temporal structures, particularly the hippocampus and adjacent limbic regions. However, the evidence synthesized in the present review increasingly supports a broader network-based model involving distributed cortico-subcortical dysfunction (Bernhardt et al. 2012; Whelan et al. 2018). Across structural, diffusion-based, functional, and electrophysiological studies, thalamic abnormalities consistently emerged as a recurrent feature of temporal lobe epilepsy. Importantly, these alterations extended beyond isolated hippocampal degeneration and frequently involved widespread disruptions of thalamocortical organization, network synchronization, and large-scale functional integration.

Large multicenter investigations, including ENIGMA-Epilepsy studies, further reinforced the concept that TLE is associated with bilateral subcortical and cortical abnormalities extending beyond the epileptogenic temporal lobe (Whelan et al. 2018). Similarly, graph-theoretical and connectivity studies demonstrated altered hub organization and reduced modular segregation involving thalamic subnetworks (He et al. 2020; Chen et al. 2024).

Collectively, these findings suggest a predominantly hippocampocentric framework toward a distributed systems-level model in which the thalamus contributes to seizure propagation, synchronization, and network-level dysfunction (Guye et al. 2006; Whelan et al. 2018).

### Nucleus-specific thalamic vulnerability

One of the most consistent observations emerging from the reviewed literature is that thalamic involvement in TLE is not homogeneous. Instead, distinct nuclei appear to demonstrate preferential vulnerability according to their anatomical connectivity and functional network affiliations. The nucleus-specific findings emerging across structural, functional, and electrophysiological studies are summarized in Table 2.

The anterior thalamic nucleus (ANT) was most consistently associated with seizure propagation and temporolimbic synchronization. Structural atrophy, reduced hippocampus–ANT connectivity, and early recruitment during seizure evolution collectively suggest a central role for the ANT within epileptic limbic circuits (Li et al. 2021; Soulier et al. 2023; Yildirim et al. 2025). These findings are particularly relevant given the increasing clinical interest in anterior thalamic neuromodulation strategies.

In contrast, the mediodorsal nucleus (MD) appeared more closely associated with higher-order cognitive and executive network dysfunction. Several studies linked MD abnormalities with impaired prefrontal integration and altered large-scale network organization, suggesting that thalamic dysfunction in TLE may contribute not only to seizure generation but also to cognitive impairment (He et al. 2020; Pang et al. 2024).

The pulvinar demonstrated partially distinct electrophysiological and functional characteristics. Evidence suggesting involvement during seizure termination and posterior cortical synchronization may indicate a modulatory role distinct from the propagation-related activity observed in the ANT (Zhang et al. 2025; Li et al. 2021).

Evidence from nucleus-specific functional imaging suggests that thalamic dysfunction in TLE is not homogeneous. Whereas anterior thalamic nuclei appear predominantly involved in memory-related limbic circuits, pulvinar and central lateral nuclei are preferentially associated with visuospatial-attentional and arousal networks, respectively (Li et al. 2021; Wills et al. 2021). These observations collectively support the hypothesis that nucleus-specific thalamic dysfunction contributes to different components of epileptic network organization, including seizure propagation, synchronization, cognitive dysfunction, and compensatory reorganization.

**Table 2:** Multimodal evidence across thalamic nuclei in temporal lobe epilepsy

Nucleus	Structural MRI	Connectivity	Electrophysiology	Main clinical association	Key references
ANT	↓ Volume/atrophy	↓ Hippocampal connectivity	Early seizure recruitment	Memory impairment; seizure propagation	(Guye et al. 2006, Li et al. 2021, Keller et al. 2014, Soulier et al. 2023, Mueller et al. 2009, Yildirim et al. 2025, Tung et al. 2022, 2023, Chiosa et al. 2017, Li et al. 2022, Assenza et al. 2020)
MD	↓ Volume	Altered prefrontal connectivity	Abnormal synchronization	Executive dysfunction	(Bernhardt et al. 2012, Pang et al. 2024, Tung et al. 2022, 2023, Chiosa et al. 2017)
Pulvinar	Variable atrophy	↓ Occipital connectivity	Seizure propagation/termination	Visuospatial deficits	(Bernhardt et al. 2012, Zhang et al. 2025, Soulier et al. 2023, Tung et al. 2022, 2023, Kim et al. 2010, Li et al. 2022, Barron et al. 2014)
CL	Limited evidence	Altered arousal network connectivity	Altered awareness-related activity	Attention/ arousal	(Li et al. 2021, Assenza et al. 2020, Wills et al. 2021)
Intralaminar nuclei	Sparse evidence	Altered network integration	Seizure recruitment	Impaired awareness	(Tung et al. 2022, 2023, Wills et al. 2021)

### TLE-MTS versus MRI-negative TLE: distinct network phenotypes

An important finding emerging from the reviewed literature concerns the apparent differences between temporal lobe epilepsy with mesial temporal sclerosis (TLE-MTS) and MRI-negative temporal lobe epilepsy (TLE-MRIneg).

TLE-MTS generally demonstrated more extensive structural degeneration involving the anterior thalamic nucleus, mediodorsal nucleus, and associated thalamotemporal pathways. These alterations were frequently associated with hippocampal atrophy, disease duration, and widespread limbic network disruption, suggesting progressive network-level degeneration extending beyond mesial temporal structures (Mueller et al. 2009; Keller et al. 2014; Yildirim et al. 2025).

By contrast, MRI-negative TLE appeared characterized by more subtle and heterogeneous abnormalities. Structural alterations were generally less extensive, whereas functional and network-level disturbances remained detectable despite the absence of overt mesial temporal sclerosis (Mueller et al. 2009; Výtvarová et al. 2016). These findings raise the possibility that TLE-MTS and TLE-MRIneg may represent partially distinct network phenotypes rather than different stages of a single pathological continuum. While TLE-MTS may involve more pronounced structural degeneration, MRI-negative TLE may preferentially involve functional dysregulation and altered network synchronization.

Nevertheless, available evidence remains limited by relatively small sample sizes and methodological heterogeneity. Further multimodal longitudinal investigations are therefore required to clarify whether these phenotypes reflect distinct pathophysiological mechanisms or variable expressions of shared network dysfunction.

### Sources of heterogeneity across studies

Despite substantial convergence across modalities, important inconsistencies remain within the current literature.

Several methodological factors likely contribute to variability across studies, including differences in imaging acquisition protocols, tractography methods, thalamic parcellation strategies, graph-theoretical pipelines, and electrophysiological recording approaches. Variability in patient populations additionally represents an important source of heterogeneity, particularly regarding seizure lateralization, disease duration, seizure frequency, medication exposure, and surgical history (Whelan et al. 2018; Keller et al. 2014).

Differences in thalamic segmentation methods may be particularly relevant. Earlier volumetric investigations frequently evaluated the thalamus as a single structure, whereas more recent high-resolution parcellation studies enabled nucleus-specific analyses revealing distinct patterns of vulnerability across anterior, mediodorsal, pulvinar, and intralaminar nuclei.

The coexistence of increased and decreased functional connectivity across studies may similarly reflect dynamic interactions between pathological synchronization and compensatory network reorganization. Functional abnormalities in TLE are unlikely to represent static processes and may vary according to disease stage, seizure burden, and cognitive reserve.

These methodological and biological sources of heterogeneity currently limit direct comparability across studies and reinforce the need for standardized multimodal approaches in future investigations.

### Clinical implications and future directions

The findings reviewed here may have important implications for future network-based approaches to temporal lobe epilepsy.

First, increasing evidence supporting nucleus-specific thalamic involvement may contribute to improved characterization of epileptic network phenotypes. Structural and functional thalamic alterations could potentially inform future investigations aimed at identifying imaging or connectivity markers relevant to disease stratification and treatment planning.

Second, the recurrent involvement of the anterior thalamic nucleus across modalities provides additional mechanistic support for neuromodulatory strategies targeting thalamic circuits (Li et al. 2021; Soulier et al. 2023). However, current evidence remains insufficient to establish definitive nucleus-specific biomarkers or therapeutic targets, and additional longitudinal studies are required before direct clinical translation.

Third, the reviewed literature highlights the importance of integrating multimodal approaches combining structural MRI, diffusion imaging, electrophysiology, and network analyses. Such strategies may improve understanding of how structural degeneration, functional dysregulation, and electrophysiological synchronization interact within epileptic networks.

Future studies would particularly benefit from:

- standardized thalamic parcellation methods;
- longitudinal postoperative designs;
- larger multicenter cohorts;
- integration of electrophysiological and neuroimaging data;
- direct comparisons between TLE-MTS and MRI-negative TLE.

### Limitations

Several limitations should be considered when interpreting the findings summarized in this review. First, substantial methodological heterogeneity exists across included studies regarding imaging modalities, electrophysiological techniques, analytical pipelines, and patient selection criteria. This heterogeneity limited direct quantitative comparison and precluded formal meta-analytic synthesis. Second, relatively few studies provided detailed nucleus-specific characterization of thalamic abnormalities, particularly in MRI-negative temporal lobe epilepsy and postoperative populations. Third, most available investigations were cross-sectional in design, limiting conclusions regarding the temporal evolution of thalamic dysfunction and its relationship to disease progression or seizure control. Finally, variability in seizure lateralization, medication exposure, disease duration, and surgical history may have contributed to inconsistencies across studies.

Despite these limitations, convergent findings across multiple modalities support a central role for thalamocortical dysfunction within distributed epileptic networks in temporal lobe epilepsy.

## Conclusion

The evidence reviewed here supports the view that temporal lobe epilepsy is a distributed network disorder involving nucleus-specific thalamic dysfunction rather than an isolated mesial temporal pathology. Across structural, functional, and electrophysiological modalities, the anterior thalamic nucleus, mediodorsal nucleus, pulvinar, and intralaminar nuclei emerged as key components of thalamocortical networks implicated in seizure propagation, cognitive dysfunction, and network reorganization. Differences between TLE-MTS and MRI-negative TLE further suggest the presence of distinct network phenotypes. Although methodological heterogeneity and limited nucleus-specific studies remain important challenges, current findings highlight the relevance of multimodal approaches for understanding thalamocortical dysfunction and may inform future network-based investigations in temporal lobe epilepsy.

## Data Availability

No datasets were generated or analysed during the current study.
